# Assessment of personal exposure to particulate air pollution: the first result of City Health Outlook (CHO) project

**DOI:** 10.1186/s12889-019-7022-8

**Published:** 2019-06-07

**Authors:** Lu Liang, Peng Gong, Na Cong, Zhichao Li, Yu Zhao, Ying Chen

**Affiliations:** 1 Department of Geography and the Environment, University of North Texas, 1155 Union Circle, Denton, TX 76203 USA; 2 Ministry of Education Key Laboratory for Earth System Modeling, Department of Earth System Science, Tsinghua University, Beijing, 100084 China; 3 Center for Healthy Cities, Institute for China Sustainable Urbanization, Tsinghua University, Beijing, 100084 China

**Keywords:** Public health, Participatory GIS, Urbanization, Particulate matter, Personal exposure

## Abstract

**Background:**

To mitigate air pollution-related health risks and target interventions towards the populations bearing the greatest risks, the City Health Outlook (CHO) project aims to establish multi-scale, long-lasting, real-time urban environment and health monitoring networks. A major goal of CHO is to collect data of personal exposure to particulate air pollution through a full profile that consists of a matrix of activities and micro-environments. As the first paper of a series, this paper is targeted at illustrating the characteristics of the participants and examining the effects of different covariates on personal exposure at various air pollution exposure levels.

**Methods:**

In the first campaign, volunteers are recruited to wear portable environmental sensors to record their real-time personal air pollution exposure and routes. After a web-based social media recruitment strategy, 50 eligible subjects joined the first campaign in Beijing from January 8 to January 20, 2018. The mean personal exposures were measured at 19.36, 37.65, and 43.45 μg/m^3^ for particulate matter (PM) with a diameter less than 1, 2.5, and 10 μm, respectively, albeit with the high spatial-temporal variations.

**Results:**

Unequal distribution of exposures was observed in the subjects with different sociodemographic status, travel behavior, living and health conditions. Quantile regression analysis reveals that subjects who are younger, less educated, exposed to passive smoking, low to middle household income, overweight, without ventilation system at home or office, and do not possess private vehicles, are more susceptible to PM pollution. The differences, however, are generally insignificant at low exposure levels and become evident on bad air quality days.

**Conclusions:**

The heterogeneity in personal exposure found in this the first CHO campaign highlighted the importance of studying the pollution exposure at the individual scale. It is at the critical stage to bridge the knowledge gap of environmental inequality in different populations, which can lead to great health implications.

**Electronic supplementary material:**

The online version of this article (10.1186/s12889-019-7022-8) contains supplementary material, which is available to authorized users.

## Background

Worldwide, cities hold the key to health management [1], especially in contemporary China. The speed of urbanization in China is unprecedented. More than 50% of total population were attracted to cities since 2011 [2] and is projected to rise to 71% in 2030 [3]. In contrast, the air quality standards in most cities in China can hardly meet the needs of urban residents. Among the various health implications that urban expansion has brought [4], air pollution is the leading environmental risk factor for death [5, 6]. Public and officials are primarily concerned with the fine particulate matter (PM) with a diameter of less than 10 μm, as they can bypass human mucus membrane and cause a variety of problems, such as asthma, decreased lung function, and increased respiratory symptoms [7]. Worldwide, exposure to fine particulate matter with a diameter of less than 2.5 μm (PM_2.5_) accounts for about 4.2 million premature deaths in 2016 [7].

To mitigate air pollution-related health risks and deliver more blue-sky days, large social costs are leveraged. For instance, the latest 13th Five-Year Plan of China — a policy blueprint that will shape China’s economic development over the next five years — contains a specific PM_2.5_ target to tackle urban smog for the first time in the history. Albeit the progress in alleviating the pollution level, the long persisted and well-documented inequality in air pollution exposure among different populations [8] has been largely neglected in policy design. For instance, Internet purchases data reveals that richer people are more likely to invest in expensive air filters to offset the health consequences of pollution [9]. To avoid the polarization of the citizen interests caused by an unequal distribution of the burden of pollution, it is a priority to evaluate how populations experience average exposures and exposure disparities, and ultimately target interventions towards the populations bearing the greatest risks.

However, most exposure assessment studies are non-specific because they rely on pollutant measurements at fixed-site monitoring stations as the surrogate. In reality, the personal exposure results from a dynamic process and a multiplicity of sources, such as inside buildings, in transit vehicles [10–13], and in the general urban environment, which are collectively not equivalent to the concentrations recorded at urban background monitoring sites. An inaccurate quantification of true exposure may lead to exposure misclassification [14] and considerable uncertainty in health risk estimates [15]. The availability of Global Positioning System (GPS) and wearable/portable sensors presents an enormous opportunity for personal sampling studies by tracking the air pollution exposure and time-activity patterns at the individual level in real time. This approach can reflect the significant degree of variability over space and time. The challenges, however, are the high cost of implementation and the hardness in collecting repetitive measures on the same group of the population over the term. A recent literature review revealed only 44 studies addressing personal exposure based on individuals’ trajectory [16].

Under this context, City Health Outlook (CHO) project is initiated with the long-term goal of establishing multi-scale, long-lasting, real-time urban environment and health monitoring networks. One important objective of CHO is to conduct spatiotemporal personal exposure assessment that allows for a realistic appraisal of the risks the populations are facing. Here, we report our first efforts in determining personal exposure using wearable sensors in the megacity of Beijing under the auspices of the CHO project. As the first paper of a series, the aim of this paper is to illustrate the characteristics of the participants and examine the effects of different covariates on personal exposure at various air pollution exposure levels. This paper begins with an introduction of the CHO project by overviewing its main objectives in Section 2 and explaining the study protocol in Section 3. In section 4 and 5, we reported and discussed the results of the first campaign on air pollution inequality.

## Methods

### A brief overview of CHO

Founded in January 2017, CHO brings together researchers from multiple disciplines to promote and assess the human health impacts of air pollution in China. A key outcome of CHO will be an established protocol for human exposure assessment that high compliance in sensor validation, personal sampler wearing, data retrieval, and validation can be achieved among different experiments. Beijing is chosen to implement the first few pilot campaigns for a protocol test, considering its pressing urban health challenges, residents’ high environmental awareness, and location convenience. Other cities in China will be gradually included to provide good representativeness of different urban environments. Through the large-scale implementation of citizen-engaged surveys and campaigns, CHO intends to increase participation by residents, the private sector, non-governmental organizations, and community groups in health management, which is recommended as a new, human-centered urbanization strategy to protect human health [1].

### Environmental monitoring instrument

**Fig. 1:**
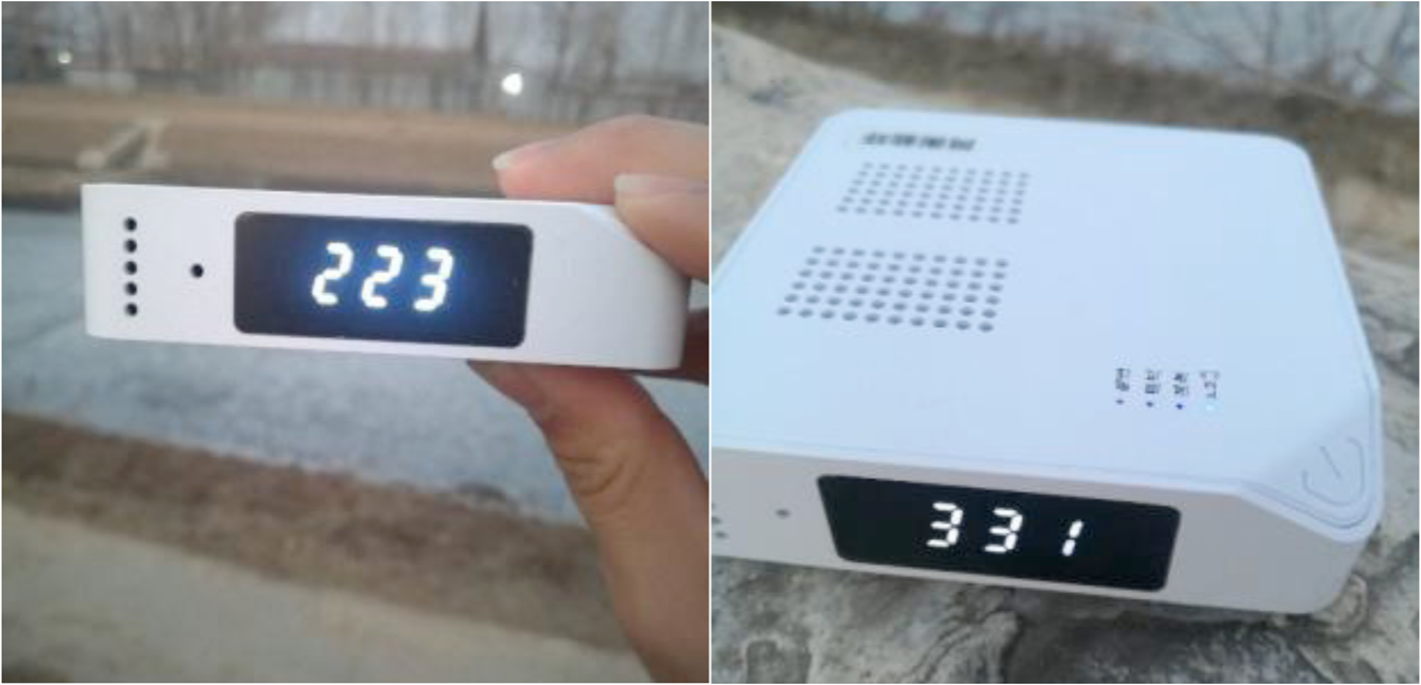
Portable environmental monitoring device TE-STR

TE-STR (Tongheng Energy & Environment Technology Institute, Beijing, China) is a portable environmental monitoring device, which has an aerosol nephelometer, a GPS receiver, a humidity, and temperature sensor built in a 90 mm × 90 mm × 22 mm box with a weight of 150 g (Fig. 1). Those sensors record the PM_1_, PM_2.5_, and PM_10_ concentrations, temperature, and humidity at 1 min sampling interval and track the movement trajectory of carriers with a GPS receiver at 5 s sampling interval. All logged data can be wirelessly transmitted to the CHO platform every 30 mins using the integrated 4G model.

The measurement accuracies of TE-STR at different PM concentrations were calibrated in the Center for Building Environment Test at Tsinghua University. The test laboratory employs a 3 m^3^ dust generation chamber, with a thorough cleaning and inspection conducted prior to calibration to ensure clean optics, good-working mechanical factors, and proper air flow rate. The TE-STR units were subjected to a TSI 8530 DustTrak II aerosol monitor test in the chamber at six different PM_2.5_ concentrations. For most applications, DustTrak calibration would be appropriate as it represents a wide spectrum of ambient aerosols. Each test was run for six times and the relative standard deviations from the TSI 8530 measurements were recorded (Additional file 1). We also compared the measurements of three TE-STR devices simultaneously against the TSI reference in the outdoor environment (Additional file 1). The results indicate that TE-STR tends to overestimate PM_2.5_ while underestimate PM_1_ and PM_10_, and the R-squared ranged from 0.49 to 0.66.

### Web-based social media recruitment

**Fig. 2:**
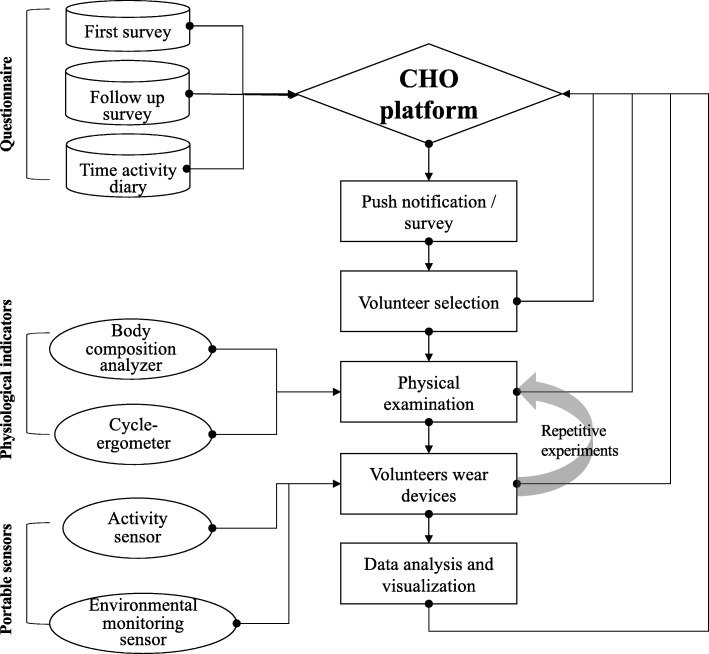
City Health Outlook Project study protocol

We recruited healthy adults in Beijing using an opportunistic recruitment approach (Fig. 2). Our recruitment advertisement was posted on several of the most influential web-based social networks of China, including Tencent WeChat, Sina Weibo, and Baidu Baijia. The number of active users of WeChat and Weibo is approximately 963 million and 340 million according to the Chinese company’s first quarter results in 2018. The online application forms were distributed through project webpage and WeChat - a cross-platform communication service (Additional file 2). The interested applicant was asked to answer 12 questions highlighting their sociodemographic characteristics, travel behavior, and health conditions. We purposely kept the first questionnaire short to engage a large candidate pool. This social media promotional strategy turned out to be successful, with over 20,000 times read and 786 applications received.

### Subject screen and training

A more comprehensive follow up survey was conducted with the 786 applicants to collect detailed information on sociodemographic characteristics (education, marital status, income), travel behavior (transport mode, private vehicle ownership), living conditions (ventilation system, passive smoking), geolocations (home, work), commute route and time, and self-reported physician diagnosis of common chronic diseases (Additional file 3). Our first screening was then set up on the basis of the inclusion criteria, with the major determiners being healthy people who are “not affected by cardiovascular disease”, “non-smokers in the age range of 20-40 years”, “drinking no more than 3 times per week”, “will live in Beijing for the next two years”, and “full time workers”. A total of 269 eligible applicants were further invited to take their physical examination at Tsinghua University and 205 applicants attended.

Our second screen was primarily based on the results of the cardiopulmonary function. Guided and supervised by professionals from Peking union medical college hospital, we tested the cardiopulmonary function, recorded blood pressure and body mass index (BMI) for each subject following a standardized procedure. Of 73 subjects (36 males and 37 females) whose cardiopulmonary function is normal, 50 finalists (25 males and 25 females) were selected for the first campaign based on their willingness in participating in multi-round campaigns and geographic locations of home and working places. Details on how the physical examination was performed are illustrated in Additional file 4.

The finalists were invited to Tsinghua on January 7, 2018 for half-day onsite training. Subjects were given details about study procedures and asked to sign consent. Immediately after the training, subjects were asked to start wearing the devices. This study complied with Tsinghua University’s guidelines regarding the participation of human subjects in research.

### Campaign and post-campaign

As our campaign completed on January 20, 2018, all devices were mailed back with prepaid shipping labels. Six subjects encountered device replacements and three subjects traveled out of Beijing for a short period. During the campaign period, each subject was asked to fill out a daily activity diary, in order for us to validate the subjects' travel routine (Additional file 5). After one week, the health examination reports and customized environment and health analytical reports were presented to each subject to promote recruitment and retention. Timely feedback to subjects’ activities is also believed to ensure the quality of data collection.

### CHO platform

All logged data can be wirelessly transmitted to the CHO platform every 30 mins using the integrated 4G model. Moreover, the platform simplifies the web-based social media recruitment by automatically sending the questionnaires to volunteers and receiving their feedback. The platform can also monitor the number of on-line devices that could help to ensure the integrity of our data. The detailed information of CHO platform was presented in Additional file 6.

### Statistical analysis

With the collected personal exposure data, two types of statistical analysis were conducted to analyze the personal exposure heterogeneity among groups that can be broadly classified into four categories: separately sociodemographic status, travel behavior, living conditions, and health status.

First, we calculated the descriptive statistics (i.e., mean and standard deviation) for the subjects’ exposure to PM_2.5_, PM_10_, and PM_1._ We further conducted analysis of variance (ANOVA) tests of mean personal exposure for different groups to analyze whether the population means of several groups are different.

Second, we introduced quantile regression to examine the effects of different covariates on personal exposure at various air pollution exposure levels. Although this method has been widely adopted in a broad spectrum of fields [17–19], to the best of our knowledge, it has not been applied in personal air pollution exposure research. The previous studies have commonly used standard linear regressions established on the assumption that the average covariate effect of the predictors on the conditional means of the response is constant. We will demonstrate that such an assumption is highly disputable and conceals the comprehensive picture of the relationship between an outcome variable and an input variable [20].

An ensemble of conditional quantile functions was analyzed by fitting separate bivariate models between individual exposure and nine characteristics (age, education, income, commute time, vehicle possession, smoking, ventilation system, BMI, respiratory disease) for quantile levels 0.1 to 0.9 at the interval of 0.05. Bootstrapping is used to estimate standard errors and confidence intervals, accounting for the hierarchical data structure [21]. The coefficients, which are interpreted as the impact of a one-unit change of the covariate on the personal exposure (μg/m^3^) while holding all other variables constant, will be compared against those derived from the ordinary least square (OLS) regression. Since the OLS coefficient remains constant across quantiles, the OLS coefficient will be plotted as a flat line with the confidence interval as two horizontal lines around the coefficient line. If the quantile coefficients fall outside of the OLS confidence intervals, they are significantly different from the OLS coefficients, and vice versa.

## Results

### Characteristics of the study population

#### Sociodemographic characteristics

Of the 50 subjects, their mean age is 30 years old and the female population is on average two years younger than the male (Table 1). The subjects are highly educated with 40% received post-graduate degree and 96% gained full-time employment. The number of unmarried subjects almost double the married ones. Middle-high income class family accounts for 68% of the subjects.

**Table 1:** Characteristics of the study population (% (N)) and the hypothesis of their effects on air pollution exposure

	Male	Female	Total	
	50% (25)	50% (25)	100% (50)	
SOCIODEMOGRAPHIC				
Age				Younger adults inhale more pollution than older people, as their activity intensity and metabolic rate is higher. And young people generally care less about self-protection.
≤ 30	48% (12)	76% (19)	62% (31)	
> 30	52% (13)	24% (6)	38% (19)	
Education				Individuals who received a higher education have higher perceptions of air pollution and are more likely to take proper actions to limit personal exposure to ambient air pollution.
Bachelor’s degree or below	60% (15)	40% (10)	50% (25)	
Post-graduate degree	40% (10)	60% (15)	50% (25)	
Marital status				Single individuals tend to engage more in outdoor activities and spend more time on leisure activities than do married individuals, which put them at a higher risk of air pollution exposure.
Single	48% (12)	84% (21)	66% (33)	
Married	52% (13)	16% (4)	34% (17)	
Annual income, RMB				Low-income individuals are more susceptible to pollution threats because of the lack of self-protective equipment, longer-distance travel, and worse working and living environment.
Low-middle: < 150,000	32% (8)	36% (9)	34% (17)	
Middle-high class: > 150,000	68% (17)	64% (16)	66% (33)	
TRAVEL BEHAVIOR				
Commute time to work, hours				Longer duration commuters increase their exposure time to unfiltered air contaminants.
≤ 1	60% (15)	68% (17)	64% (32)	
> 1	40% (10)	32% (8)	36% (18)	
Own vehicle, yes	40% (10)	20% (5)	30% (15)	Individuals in vehicles are likely to be exposed to more pollution since a car in traffic takes in and trap pollution from the exhaust of vehicles in front of it. Pedestrians and cyclists have diluted pollutants due to better airflow.
LIVING CONDITION				
Days suffered from passive smoking for more than 15 mins per week				Individuals who are exposed to secondhand smoke are more likely to inhale more pollutants.
0	72% (18)	56% (14)	64% (32)	
> 1	28% (7)	44% (11)	36% (18)	
Have ventilation system at home or office	56% (14)	40% (10)	48% (24)	The air cleaning effect of ventilation system will lower the concentration of indoor air pollutants.
HEALTH STATUS				
Body mass index, kg/m^2^				Overweight or obese adults breathe more air per day than an adult with a healthy weight, which makes them more vulnerable to air contaminants.
Normal (< 25)	68% (17)	84% (21)	76% (38)	
Overweight / Obese(≥25)	32% (8)	16% (4)	24% (12)	
Respiratory diseases	12% (3)	16% (4)	14% (7)	Patients with respiratory diseases are more likely to be cautious about bad air quality days and tend to take more protections.

#### Travel behavior

**Fig. 3:**
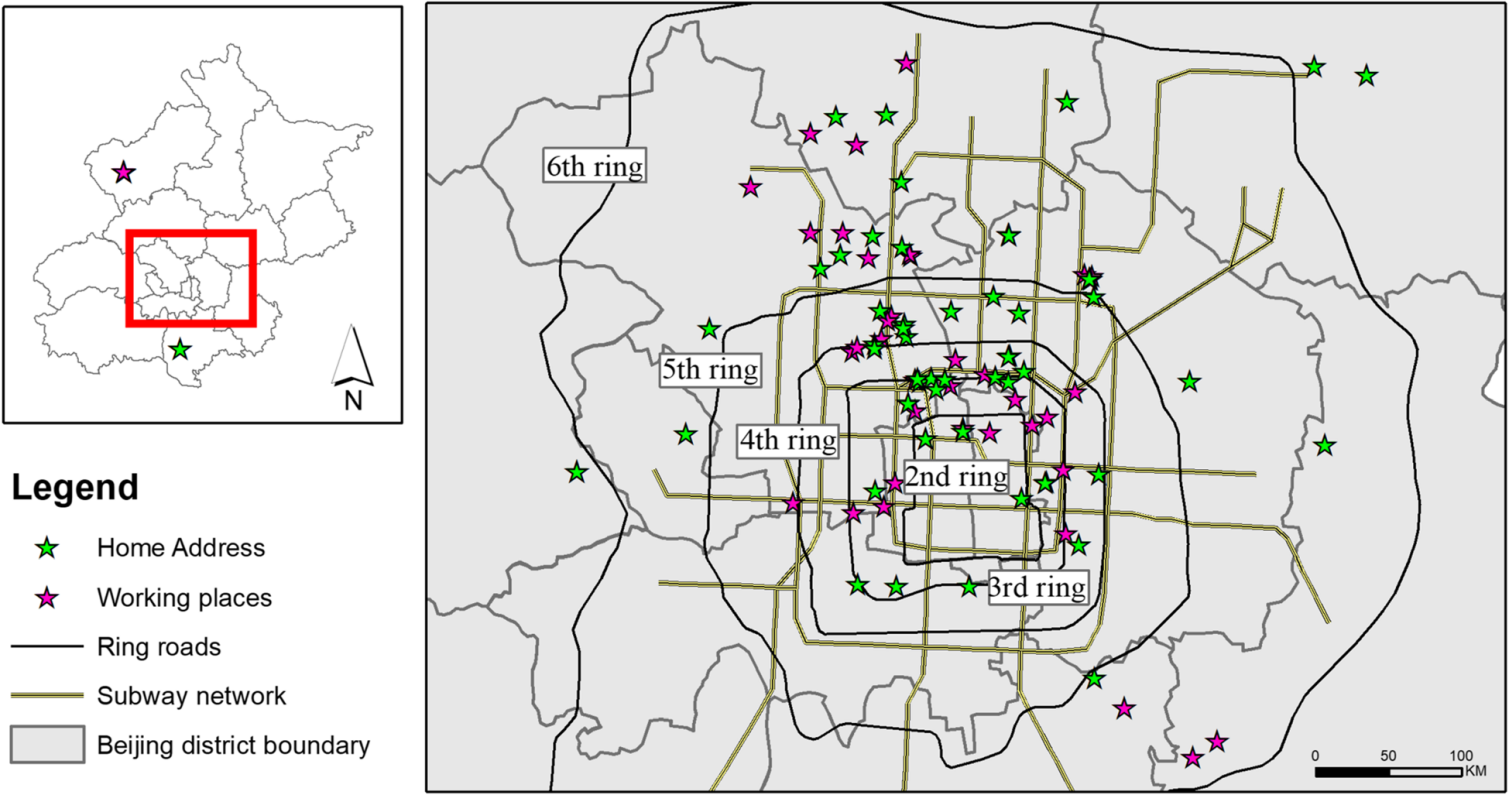
Distribution of the 50 finalists’ primary home and working address in Beijing during our first campaign. Data sources: ring road, subway network, and Beijing district boundary data were obtained from OpenStreetMap

The subjects’ home and office locations spread over the urban part of Beijing. Except for five subjects who live out of the 6th ring road, all the others reside within the 6th ring road (Fig. 3). Their residential addresses cover 12 out of 16 Beijing’s districts and their working places are distributed in nine districts. Sixty percent of the subjects spent less than an hour to commute from home to work. The share of subjects with a private vehicle is 30%.

#### Living condition

Sixty-four percent of the subjects do not suffer from passive smoking for more than 15 mins per week, with more females than males. The share of subjects with ventilation system installed at home or office is 48%.

#### Health status

Thirty-eight subjects’ body mass index is within the normal range, but 12 of them are indicated as overweight. The prevalence of the respiratory disease is 12% in males and 16% in females. No one reported being diagnosed with cardiovascular disease.

### Descriptive statistics of personal exposure in different groups

The average personal exposure for all 50 subjects was 19.36, 37.65, and 43.45 μg/m^3^ for PM_1_, PM_2.5_, and PM_10_, respectively. Using 25 and 50 μg/m^3^ as the reference concentrations for PM_2.5_ and PM_10_ established by the World Health Organization (WHO) air quality guidelines [22], the personal exposure is much higher for the recommended fine PM level and slightly lower than that of coarse PM. The findings on all three types of PM were similar and will not be particularly mentioned afterwards.

Except for the gender and marital status, significant differences in personal exposure were observed (Table 2). The younger subject group with age under 30 had the higher mean personal exposure. Subjects with the highest education in a bachelor’s degree or below were exposed to more air pollution than those received post-graduate degree. The standard deviation, which quantifies the differences between the lowest and the highest exposure within a particular population, shows a wider range in the lower education level group. The low-middle class experienced higher mean exposure.

**Table 2:** Statistic parameter and ANOVA tests of mean personal exposure for different groups

	Mean ± STD (μg/m^3^)		
	PM_2.5_	PM_10_	PM_1_
SOCIODEMOGRAPHIC			
Gender			*
Female	35.85 ± 56.28	41.25 ± 60.81	18.36 ± 29.07
Male	40.38 ± 55.45	46.73 ± 61.65	20.80 ± 25.70
Age	***	***	***
≤30	38.55 ± 59.36	44.41 ± 64.42	19.72 ± 29.65
> 30	37.65 ± 49.80	43.60 ± 55.94	19.48 ± 23.33
Education	***	***	***
Bachelor’s degree or below	44.40 ± 66.40	51.12 ± 71.86	22.67 ± 32.76
Post-graduate degree	31.64 ± 40.96	36.65 ± 46.47	16.40 ± 19.70
Marital status			
Single	38.93 ± 58.76	44.65 ± 63.72	19.94 ± 29.07
Married	36.98 ± 50.57	43.16 ± 56.92	19.10 ± 24.26
Annual income, RMB	***	***	***
Low-middle: < 150,000	43.64 ± 67.20	49.52 ± 71.36	22.58 ± 34.39
Middle-high class: > 150,000	35.46 ± 48.97	41.36 ± 55.34	18.14 ± 22.93
TRAVEL BEHAVIOR			
Commute time to work, hours	***	***	**
≤ 1	36.58 ± 55.20	42.08 ± 60.14	18.96 ± 28.28
> 1	41.14 ± 57.01	47.73 ± 63.19	20.83 ± 25.69
Own vehicle	***	***	***
yes	33.34 ± 48.20	38.66 ± 54.79	16.77 ± 22.67
no	40.30 ± 58.78	46.44 ± 63.77	20.86 ± 29.11
LIVING CONDITION			
Days suffered from passive smoking for more than 15 mins per week	**	***	*
0	37.06 ± 58.60	42.56 ± 63.38	19.11 ± 29.37
> 1	40.25 ± 50.65	46.83 ± 57.34	20.56 ± 23.45
Have ventilation system at home or office	***	***	***
yes	35.58 ± 57.05	40.80 ± 61.51	18.37 ± 30.03
no	40.47 ± 54.78	46.94 ± 60.99	20.71 ± 24.87
HEALTH STATUS			
Body mass index, kg/m^2^	***	***	***
Normal (< 25)	35.40 ± 53.69	40.91 ± 58.87	18.16 ± 27.08
Overweight / Obese(≥25)	46.37 ± 61.14	53.38 ± 67.03	23.91 ± 27.86
Respiratory diseases	*	**	*
yes	34.51 ± 46.60	39.67 ± 52.03	17.77 ± 21.18
no	38.21 ± 55.50	44.21 ± 61.03	19.67 ± 27.73

Note: * denotes significance level < 0.05; **significance level < 0.01; ***significance level < 0.001. The highest personal exposure concentration in each group was shaded in grey

The subjects who spend more time in their one-way commute to work (more than an hour) were exposed more. For the fifteen subjects who own private vehicles, their average PM_2.5_ exposure was 6.96 μg/m^3^ lower than those without. Although all subjects are non-smokers, those who were exposed to passive smoking for more than 15 mins per day had significantly higher exposure level than those who did not. The ventilation system reduced the exposure level, as the PM_2.5_ exposure is 4.89 μg/m^3^ lower in subjects with ventilation systems operated at home or office. The overweight population had significantly higher personal exposure than people with normal weight, and the difference is 10.97 μg/m^3^ for PM_2.5_. Subjects with self-diagnosed respiratory diseases have lower exposure level than those without.

### Quantile regression results

**Fig. 4:**
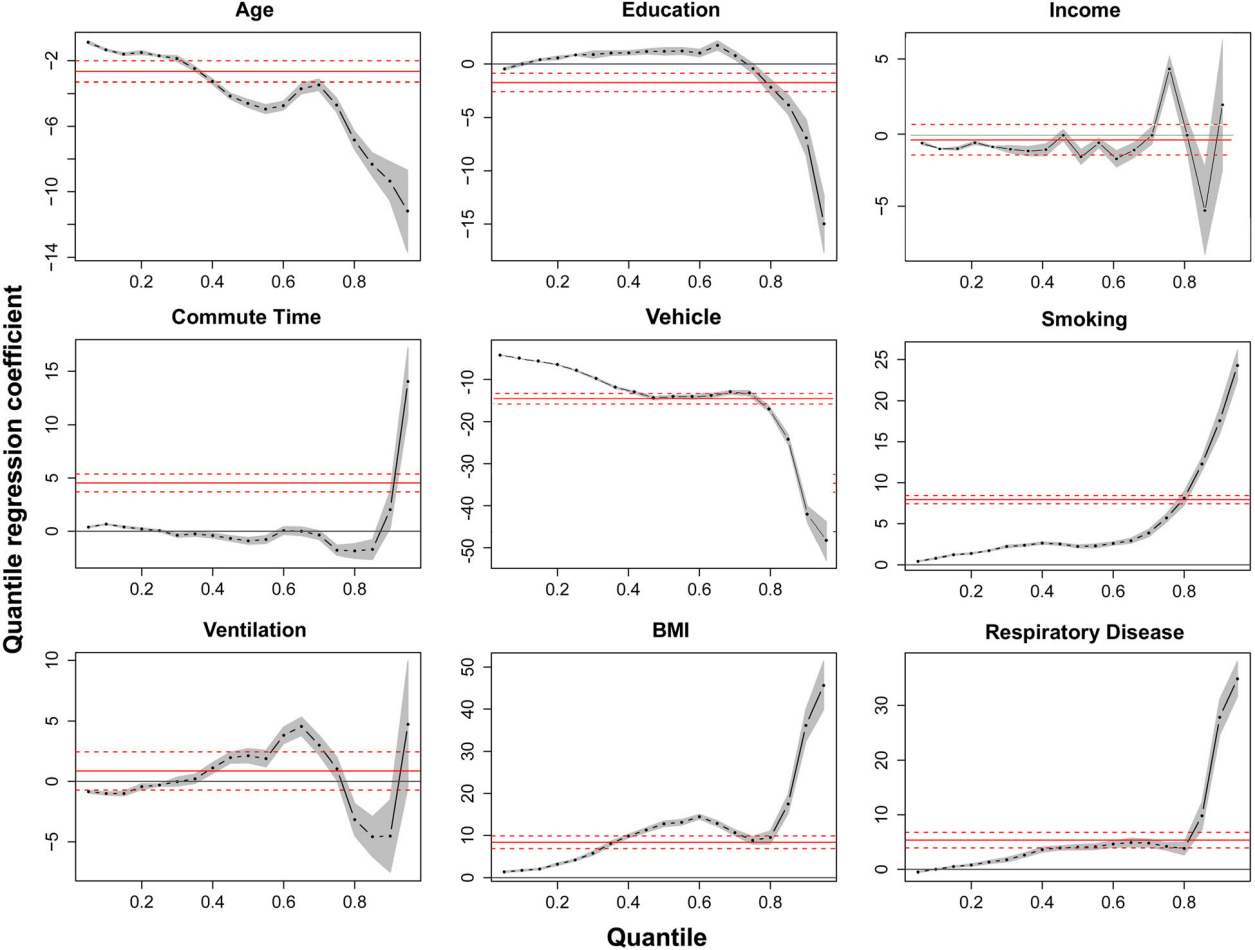
The effects of sociodemographic, travel behavior, living conditions, and health status on personal PM_2.5_ exposure. Each dot on the black lines represents quantile regression coefficients and grey shadings indicate 95% confidence intervals as a function of the quantile level. The red horizontal solid and dashed lines depict the OLS coefficient estimates and the associated 95% confidence intervals, respectively

Variables (characteristics) that showed no significant differences between groups in Table 2 were not included for quantile regression. Table 3 displays the quantile regression results at the 0.25, 0.5 0.75, and 0.9 quantiles and their comparison with OLS coefficient estimates. Figure 4 displays nine influence plots that present the relationship between personal exposure and the most revealing variables in the quantile regression model.

**Table 3:** Coefficient estimates of OLS and quantile regression at different quantiles

	OLS	Quantile			
		0.25	0.5	0.75	0.9
Age	−2.65^a^	−1.70^a^	− 4.59^a^	−4.70^a^	−9.35^a^
Education	−1.72^a^	0.85^a^	1.19^a^	−0.43	−6.90^a^
Income	−0.32	−0.53	0	−1.03^a^	−5.25^a^
Commute time	4.54^a^	0.05	−0.91^a^	−1.78^a^	2.03
Vehicle	−15.99^a^	−6.51^a^	−15.79^a^	− 14.55^a^	−48.54^a^
Smoking	7.93^a^	1.70^a^	2.22^a^	5.70^a^	17.56^a^
Ventilation	0.87	−0.30^a^	2.13^a^	1.04^a^	−4.52^a^
BMI	8.36^a^	4.15^a^	12.75^a^	8.83^a^	36.17^a^
Respiratory	5.35^a^	1.33^a^	4.06^a^	4.20^a^	27.87^a^

Note: ^a^denotes significantly different coefficient from zero at the 5% significance level

Age negatively correlates with personal exposure, especially at the upper tail of the personal exposure histogram distribution. The higher confidence limits are lower than the OLS estimate for quantile levels higher than 0.7. Younger subjects are exposed more to PM_2.5_ pollutants, and the effect at the 0.9 quantile is 5.5 times stronger than that at the 0.25 quantile. The influence of education on personal exposure is negative but only for the high quantiles, whereas no significant effect is observed on the lower quantiles. Personal exposure in different income groups did not show an obvious pattern. No significant relationship was observed until the 0.7 quantile, above which personal PM_2.5_ first increased with income, followed by an abrupt drop and a slight increase after the 0.85 quantile.

Among the subjects who commute to work at different time duration, their coefficients were not significantly different from zero before the 0.9 quantile. As approximating to the right end of exposure distribution, longer commute time dramatically elevates personal exposure. Subjects owning private vehicles generally receive less exposure at all ranks of quantiles, and the air pollution reduction effect of cars is especially prominent at high exposure levels.

The effect of passive smoking is positive and the coefficient increases exponentially after the median quantile. A high difference of 17.56 μg/m^3^ could be observed between populations who receive passive smoking and those who do not when the total exposure level is high. The effect of the ventilation system is complex, as the coefficients increased stably from the left tail to the 0.6 quantile and then started dropping till the 0.9 quantile but increased sharply at extremely far right quantile (Fig. 4).

In terms of the influence of health status on personal exposure, overweight subjects received 4.15 μg/m^3^ higher PM_2.5_ exposure at a low exposure level (at the 25% quantile) and 36.17 μg/m^3^ more at a high exposure level (at the 90% quantile). Lastly, the presence of respiratory diseases in the subjects leads to fairly constant coefficient values before the 0.8 quantile but increases towards at the distribution’s right tail.

## Discussions

### Personal exposure disparity

By testing the personal exposure differences among different sociodemographic groups, no significant difference was observed between the male and female, and the married and single in our subjects. Nonetheless, age, education, and income are prominent in influencing the level of exposure to air pollutants.

The impacts of age and education on PM exposure are not significant when the overall exposure level is low but become prominently negative as the exposure level elevates. Younger subjects experiencing elevated levels of air pollution may be attributed to their high activity intensity and extended outdoor activities. Education has been long recognized to have a profound positive impact on population health [23], which is also evident in reducing PM exposure level in our study. People with higher education are better aware of the adverse effects of air pollution and take proper self-protection actions [24], such as checking daily air quality index and avoiding outdoor activity or wearing respirators when air quality is bad.

We also noticed income inequalities in PM exposure, but there is no clear pattern on how income affects exposure as revealed by the quantile regression. Low-income subjects are most likely active commuters with the dominant transport mode as cycling, bus, light-rail train, and walking [21, 25]. Those modes with direct exposure to traffic increase the inhaled dose of air pollution [26]. But this negative relationship reversed after the 0.85 exposure quantile. It is unclear whether this is due to the small subject samples and needs further study.

#### Travel behavior

Individuals who work indoor and commute to work receive a substantial portion of their daily dose of air pollution in their working environment and during commuting activities. In our study, commute time, passive smoking, possession of a personal vehicle, and ventilation systems are presented as important factors in determining the exposure to air pollutants.

Traffic-related air pollution contributes significantly to commuters’ daily PM_2.5_ exposure [27]. Without a doubt, longer commuting time accumulates the inhaled dose, regardless of the different transport modes. A one-year aerosol characterization study in Beijing presented that the differences in PM_2.5_ concentrations on the 4th ring road were 44 μg/m^3^ higher than rural sites [28]. Nonetheless, our study reveals that differences in PM exposures across work commute time groups were small and only became obvious when high exposure level is reached.

The occupancy of private vehicles results in lower PM exposure. Although most air intake filters in cars have relatively low efficiency and pollutants can penetrate through openings such as window and door seals, with proper vehicle operating conditions and the equipment of cabin recirculation filters, the reduction of in-cabin PM exposure can still be significant [29]. Driving with the window closed is more protective against traffic-related PM exposure than other transport modes [27], especially on high pollution days.

#### Living conditions

Tobacco smoking is a major indoor PM source where smoking is permitted [30]. As expected, our results show that subjects exposed to secondhand smoking inhaled more air pollution than those who did not, although the difference is marginal (3.19 μg/m^3^). In contrast to smoking that elevates the indoor pollution level, ventilation systems reduced 4.89 μg/m^3^ exposure concentration on average. However, the air cleaning effect varied at different exposure levels, which was insignificant at low exposure levels and became evident at medium-high levels. One study reported that a ventilated classroom had PM_10_ concentrations on average 66% lower than those measured in the unventilated control classroom [31]. Nearly half of the subjects have a ventilation system installed at home or office. In China, with the increasing public awareness of air pollution, the trend of opting to purchase an air ventilation system to regulate indoor air quality will keep growing in the future.

#### Health status

Our data also suggests a lower exposure level in subjects with existing respiratory diseases. Considering their sensitivity to air pollutants, those subjects may take more effective personal interventions to decrease their susceptibility to air pollution [32]. The overweight subjects were exposed to the environment with higher ambient PM concentration. This may be attributed to the fact that overweight people are more prevalent among individuals of lower education [33], who tend to have less health awareness or live and work in environments with worse air quality.

### Health implications of air pollution inequality

The most prominent outcome of the disparity in individuals’ pollution exposure could be health inequalities, especially in individuals or communities with lower socioeconomic position [21]. Exposure to disproportionately high levels of PM can lead to various health-damaging levels. For example, the WHO suggests that a 100 μg/m^3^ increase in the daily average concentration of PM_10_ can result in a 7% increase in daily mortality and an 8% increase in daily hospital admission [34]. A 10 μg/m^3^ increase of PM_10_ was related with statistically higher risk of death of 0.64% for older populations (> = 65 years) and 0.34% for younger populations [35]. In London, a 1.1 μg/m^3^ increase in PM_2.5_ was associated with a decline in some measures of cognitive function in elderly people [19], and a 2.2 μg/m^3^ difference in PM_2.5_ may increase the likelihood of low birth weight [36]. However, most environmental inequality studies were in North America and Europe. The quantifications have not been tested widely in China. Although numerous scientific studies have shown a strong and consistent linkage of particle pollution exposure to a variety of health problems, the evidence regarding susceptibility, vulnerability, and modifying factors is inconclusive. As China is experiencing a transition of the disease patterns from infectious disease to non-communicable disease, bridging the knowledge gap of environmental inequality in different populations of China will have great health implications, such as aid design regulations that target local air quality control efforts to specific populations.

### Limitations

Although the first CHO campaign has reached its goals, there were some unavoidable limitations. The main limitation is the small subject sample size, and the subjects are healthy adults, which may hinder the interpretation of results. This is partly due to the cost of portable air-quality sensors that restricts the implementation on a large population. Although the price is much reduced, the unit we used is around USD 300. Also, considering that the campaign should be conducted by subjects during the same period to allow a fair comparison, it is quite challenging to recruit thousands of subjects at one time. Thus, conclusions drawn out of this study should be applied conservatively. One recommendation for comparing characteristics between groups of subjects in small studies is looking at the degree of difference [37]. For small differences, it is hard to determine whether the exposure difference is due to the subjects’ characteristics or simply chance. However, a large difference is unlikely to all be due to chance.

Another major limitation is what the subjects collected are ambient pollution concentration, not inhaled dose. Most subjects carried the devices in their backpacks or handbags, which measures the ambient concentration at the waist height. In epidemiological studies, the amount of pollution reaching the lungs depends on the inhalation dose, which is not only related to the ambient pollution concentration but also affected by physical activity and ventilation rates [38]. Ideally, a facemask is used to measure the dose but is uncomfortable to wear over a few days. Various methods have been proposed to estimate the inhaled dose based on physical activity type [39], energy expenditure [40], heart rate [41, 42], and breathing rate [43, 44]. In our campaign, besides the environmental sensors, subjects also carried an ActiGraph GT3X (Pensacola, Florida) accelerometer simultaneously to monitor human rest and physical activity levels. It is thus feasible to estimate the inhaled dose for individuals from sensor recorded ambient concentrations using the above methods.

A third limitation is the challenge to have low-cost sensors reach the data quality of high-end instrument. Information provision regarding low-cost sensor performance is not prevalent and just emerging [45]. The sensor manufacturer of TE-STR provided its performance data in a controlled environment, but has not evaluated the data quality and stability over long-term deployment in the field with varying environmental conditions. The differences between laboratory calibration and field performance evaluation are also witnessed in our study. It is recommended that low-cost sensor data can be used to obtain relative and aggregated information about the ambient air quality [45]. Thus, findings from this study should be used carefully, and we recommend using the relative comparison among exposure levels of different population groups instead of the absolute differences.

## Conclusion

The present study summarizes the project overview, study design, and the results of the first campaign of CHO project. The preliminary data analysis highlighted the unequal distribution of PM exposures among different populations, especially in bad air quality conditions. The limitations in sample size also suggests that future campaigns should be encouraged and findings could guide the development of protocols to increase participation in the future.

## Additional files

Additional file 1: The validation of TE-STR against TSI aerosol monitor in both chamber-controlled environment and outdoor environment. (DOCX 630 kb)

Additional file 2: The online application form used to recruit volunteers. (DOCX 270 kb)

Additional file 3: Follow up survey questions. (DOCX 24 kb)

Additional file 4: The physical examination procedure used in this study. (DOCX 15 kb)

Additional file 5: Daily activity diary that each subject was asked to fill during the survey campaign. (DOCX 18 kb)

Additional file 6: The core modules of the CHO Platform. (DOCX 16 kb)

## Data Availability

The data collected for this study is not publicly available. Request to access data sets can be made to the corresponding author.

## Abbreviations

ANOVA: Analysis of variance
BMI: Body mass index
CHO: City health outlook
GPS: Global positioning system
OLS: Ordinary least square
PM: Particulate matter
WHO: World Health Organization
