# Supplementary material for: Assessment of personal exposure to particulate air pollution: the first result of City Health Outlook (CHO) project
Source: BMC Public Health. 2019 Jun 7;19:711. doi: 10.1186/s12889-019-7022-8 (PMC6555980; doi:10.1186/s12889-019-7022-8)
Supplement: Supplementary file 1 — The validation of TE-STR against TSI aerosol monitor in both chamber-controlled environment and outdoor environment. (DOCX 630 kb) [file 12889_2019_7022_MOESM1_ESM.docx]

**Additional file 1**. The validation of TE-STR against TSI aerosol monitor in both chamber-controlled environment and outdoor environment.

**Table S1**. Accuracy test of TE-STR against TSI 8530 DustTrak II aerosol monitor for PM_2.5_ measurements.

| TSI 8530 (μg/m^3^) | TE-STR002GPS measurements (μg/m^3^) | | | | | | RSD (%) |
| --- | --- | --- | --- | --- | --- | --- | --- |
|  | Test 1 | Test 2 | Test 3 | Test 4 | Test 5 | Test 6 |  |
| 4 | 5 | 5 | 6 | 5 | 6 | 7 | 13.15 |
| 49 | 51 | 51 | 51 | 51 | 50 | 52 | 1.13 |
| 98 | 103 | 102 | 102 | 105 | 94 | 99 | 3.5 |
| 124 | 132 | 129 | 127 | 134 | 113 | 125 | 5.37 |
| 188 | 139 | 179 | 143 | 161 | 162 | 160 | 8.42 |
| 427 | 383 | 429 | 396 | 397 | 377 | 396 | 4.15 |

Note: RSD – relative standard deviation.


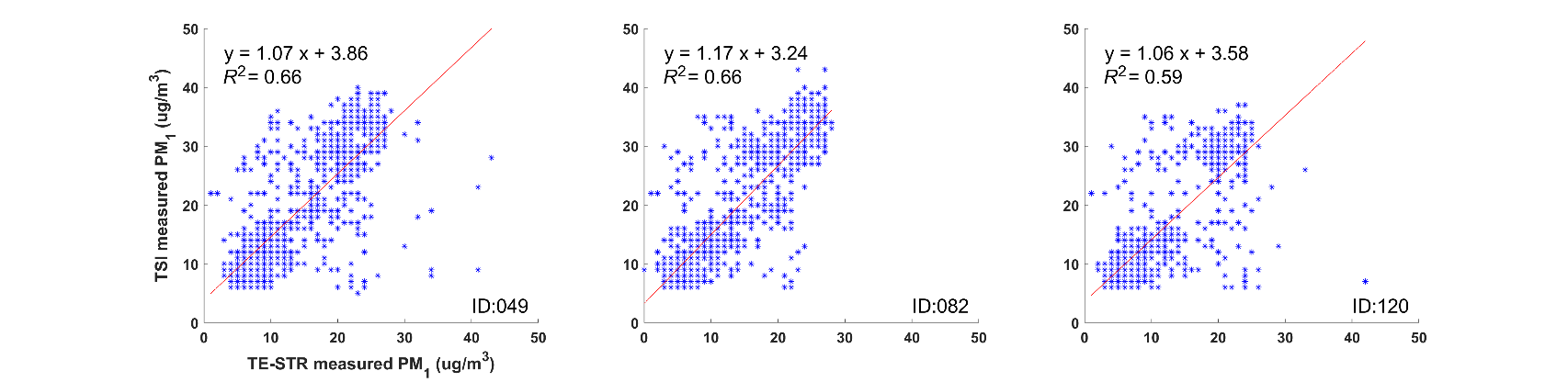
a)

b)


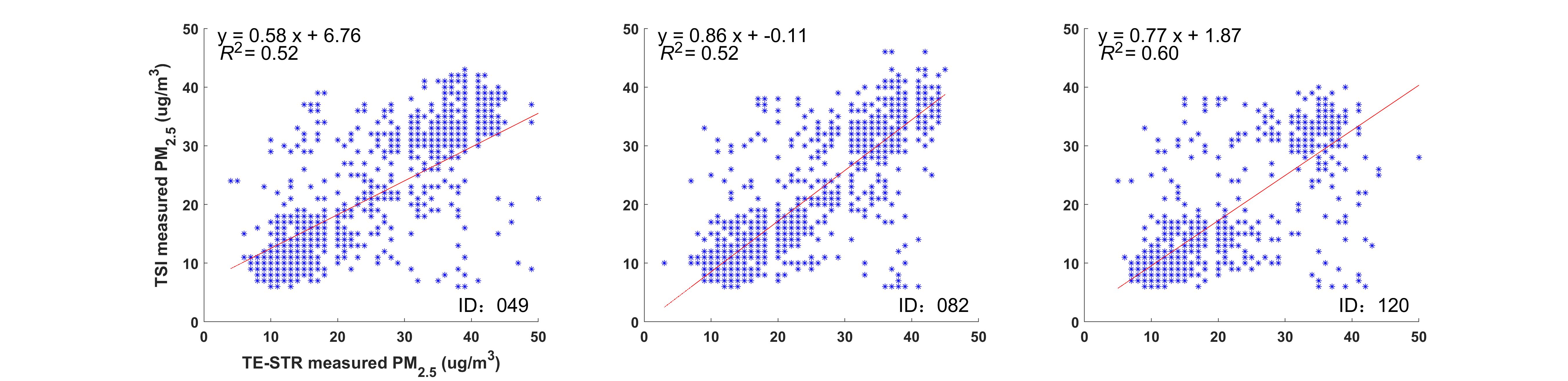


c)


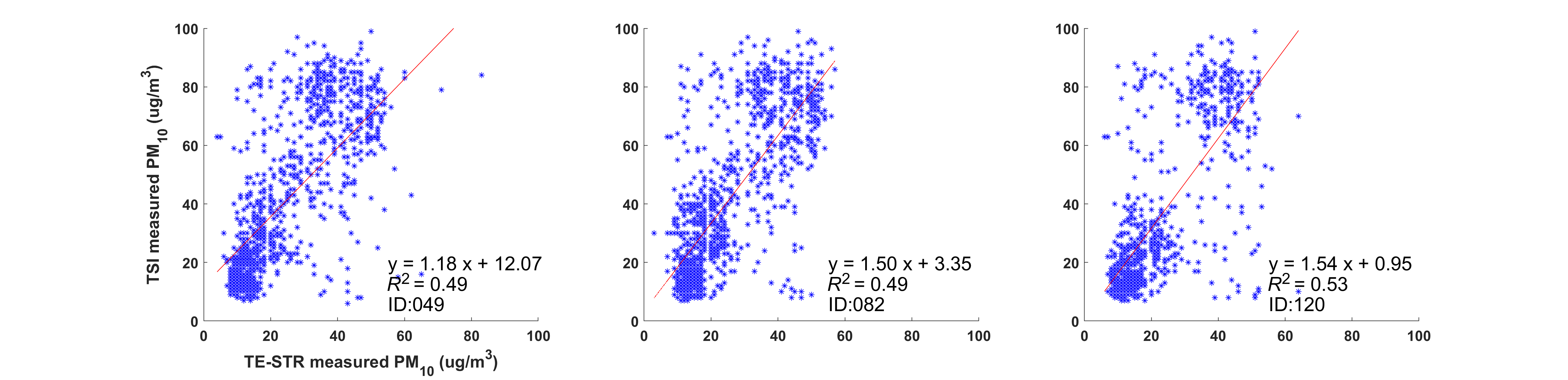


**Figure S1**. The scatter plots showing the measured values for a) PM_1_, b) PM_2.5_ and 3) PM_10_ of three TE-STR devices on the *x-axis*, against the reference TSI 8530 DustTrak II aerosol monitor on the *y-axis*.
