# Supplementary material for: Assessment of personal exposure to particulate air pollution: the first result of City Health Outlook (CHO) project
Source: BMC Public Health. 2019 Jun 7;19:711. doi: 10.1186/s12889-019-7022-8 (PMC6555980; doi:10.1186/s12889-019-7022-8)
Supplement: Supplementary file 2 — The online application form used to recruit volunteers. (DOCX 270 kb) [file 12889_2019_7022_MOESM2_ESM.docx]

**Additional file 2.** The online application form used to recruit volunteers.

1) Gender

○ Male

○ Female

2) Age

○ 20 and below

○ 21-25

○ 26-30

○ 31-35

○ 36-40

○ 41 and above

3) Your most recent height and weight

_______cm， ________kg

4) Currently, how many family members are you living with? _______

Do you have any child below 3 years old living with you?

○ Yes

○ No

5) your current employment status

○ full time

○ part time

○ seeking employment

○ retired

○ students

○ other _________

6) Where do you work?

○ home office

○ unfixed place of work

○ stationary office

7) How long have you been working？

○ none

○ < 5 years

○ 6-10 years

○ 11-15 years

○ > 16 years

8) Will you be working and living in Beijing in the next two years?

○ Yes

○ No

9) Which of the following transportation method is used most frequently by you on a weekly basis (Fill the circles if appropriate)

|  | Never | 1-3 days | 4-5 days | 6-7 days |
| --- | --- | --- | --- | --- |
| Subway | ○ | ○ | ○ | ○ |
| Bus | ○ | ○ | ○ | ○ |
| Taxi or you drive your own car | ○ | ○ | ○ | ○ |
| Electric/motorcycle | ○ | ○ | ○ | ○ |
| Bike | ○ | ○ | ○ | ○ |
| Walk | ○ | ○ | ○ | ○ |

10) Check all the districts that are your main activity areas (e.g., work, living, entertainment etc.) (could choose more than one)

□ Dongcheng district

□ Xicheng district

□ Haidian district

□ Chaoyang district

□ Tongzhou district

□ Shunyi district

□ Changping district

□ Daxing district

□ Fengtai district

□ Fangshan district

□ Shijingshan district

□ Mentougou district

□ Huairou district

□ Pinggu district

□ Yanqing district

□ Miyun district

11) Check all the options that apply to you？

□ smoked

□ drunk for more than 3 times a week

□ suffered from cardiovascular diseases

□ suffered from chronic respiratory diseases

□ none of the above

12) These are the devices that you will be wearing in this study. Are you willing to participate?


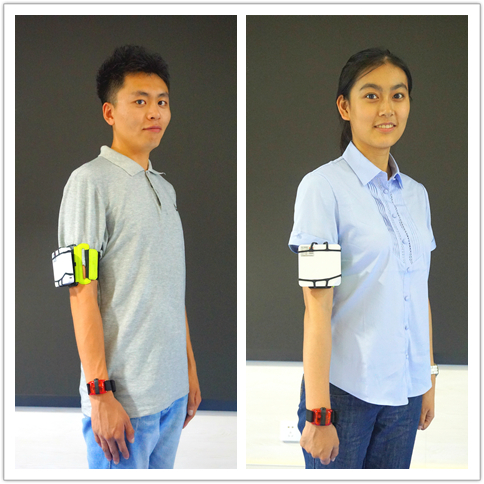


○ Yes

○ No
