# Supplementary material for: Assessment of personal exposure to particulate air pollution: the first result of City Health Outlook (CHO) project
Source: BMC Public Health. 2019 Jun 7;19:711. doi: 10.1186/s12889-019-7022-8 (PMC6555980; doi:10.1186/s12889-019-7022-8)
Supplement: Supplementary file 3 — Follow up survey questions. (DOCX 24 kb) [file 12889_2019_7022_MOESM3_ESM.docx]

**Additional file 3.** Follow up survey questions.

1) Highest level of education achieved

○ High school or below

○ Bachelor

○ Master

○ PhD

2) Marital status

○ Single

○ Married

3) Your ethnicity group

○ Han

○ Manchurian

○ Hui/Muslim chinese

○ Mongolian

○ Korean/ Chaoxian

○ Du

○ Zhuang

○ Miao

○ Other ______________

4) Ancestral hometown_____________

5) How long have you been living in Beijing ( ) years

6) You are currently live in?

○ owned house

○ rental room

○ unit apartment

○ other ___________

7) Do you own any vehicle?

○ Yes

○ No

8) Please provide us your home address ___________

9) Please provide us your working address ___________

10) Are there any ventilation system or air purification devices installed at your home or working places?

○ Yes

○ No

11) Typically, how long does it take you to go to work (from home to workplace)?

○ < 0.5 hour

○ 0.5 - 1 hour

○ 1-2 hours

○ 2-3 hours

○ > 3 hours

12) On which days do you need to go to work?

□ Monday

□ Tuesday

□ Wednesday

□ Thursday

□ Friday

□ Saturday

□ Sunday

13) When do you leave home for work?

○ 1:00

○ 2:00

○ 3:00

○ 4:00

○ 5:00

○ 6:00

○ 7:00

○ 8:00

○ 9:00

○ 10:00

○ 11:00

○ 12:00

○ 13:00

○ 14:00

○ 15:00

○ 16:00

○ 17:00

○ 18:00

○ 19:00

○ 20:00

○ 21:00

○ 22:00

○ 23:00

○ 24:00

14) When do you get off from work?

○ 1:00

○ 2:00

○ 3:00

○ 4:00

○ 5:00

○ 6:00

○ 7:00

○ 8:00

○ 9:00

○ 10:00

○ 11:00

○ 12:00

○ 13:00

○ 14:00

○ 15:00

○ 16:00

○ 17:00

○ 18:00

○ 19:00

○ 20:00

○ 21:00

○ 22:00

○ 23:00

○ 24:00

15) How many business trips do you typically make per month?

○ Never

○ ≤ 3times

○ ≥ 4 times

16) Typically, how long does your business trip last?

○ ≤3 days

○ 4-6 days

○ 7-9 days

○ ≥10 days

17) your most recent blood pressure

systolic pressure ____mmHg

diastolic pressure ____mmHg

18) Have you ever suffered from any cardiovascular diseases (coronary heart disease, angina pectoris, myocardial infarction, myocarditis, dilated cardiomyopathy, rheumatic heart disease, arrhythmia, heart failure, sick sinus syndrome etc.)?

○ Yes

○ No

○ Uncertain

19) Have you ever suffered from any respiratory diseases (Asthma, pneumonia, tracheitis, bronchitis, emphysema, chronic bronchitis, pulmonary heart disease, tuberculosis, chronic obstructive pulmonary disease etc.)?

○ Yes

○ No

○ Uncertain

20) Have any of your parents suffered from cardiovascular or respiratory diseases?

○ Yes

○ No

21) Have you been frequently affected by any of the following health issues?

|  | Yes | No |
| --- | --- | --- |
| Dizziness | ○ | ○ |
| Faint | ○ | ○ |
| Fever | ○ | ○ |
| Flustered | ○ | ○ |
| Chest tightness | ○ | ○ |
| Edema of the lower limbs | ○ | ○ |
| physical difficulties | ○ | ○ |
| Heartache | ○ | ○ |
| Chronic cough | ○ | ○ |
| cough with mucus | ○ | ○ |
| Pain in the chest | ○ | ○ |
| Short of breath | ○ | ○ |
| Cough with blood | ○ | ○ |

22) In one week, how many days do you suffer from passive smoking for more than 15 minutes?

○ None

○ 1 day

○ 2 days

○ 3 days

○ 4 days

○ 5 days

○ 6 days

○ 7 days

23) Within the last week, **how many days** did you experience high/moderate/low intensity physical activities for more than 10 minutes?

|  | None | 1 day | 2 days | 3 days | 4 days | 5 days | 6 days | 7 days |
| --- | --- | --- | --- | --- | --- | --- | --- | --- |
| High intensity | ○ | ○ | ○ | ○ | ○ | ○ | ○ | ○ |
| Moderate intensity | ○ | ○ | ○ | ○ | ○ | ○ | ○ | ○ |
| Low intensity | ○ | ○ | ○ | ○ | ○ | ○ | ○ | ○ |

High intensity activities refer to activities that lead to obvious increases in heart rate and breathing rate, like workout in gym, ball games, swimming etc. Moderate intensity activities refer to activities that lead to increases in heart rate and breathing rate, like jogging etc.

24) Within the last week, how long did you need to carry out high/moderate/low intensity physical activities **per day**?

|  | < 10 mins | 10-30 mins | 30-60mins | 1-1.5 hour | 1.5-2hours | 2 hours |
| --- | --- | --- | --- | --- | --- | --- |
| High intensity | ○ | ○ | ○ | ○ | ○ | ○ |
| Moderate intensity | ○ | ○ | ○ | ○ | ○ | ○ |
| Low intensity | ○ | ○ | ○ | ○ | ○ | ○ |

25) What kind of transportation do you use for the following activities?

|  | Subway | Bus | Taxi or drive your own car | Electric/motorcycle | Bike | Walk |
| --- | --- | --- | --- | --- | --- | --- |
| Work | □ | □ | □ | □ | □ | □ |
| Social gathering | □ | □ | □ | □ | □ | □ |
| Shopping | □ | □ | □ | □ | □ | □ |
| Excursion | □ | □ | □ | □ | □ | □ |

26) On a typical day,

when do you go to bed? ___________

When do you get up? __________

Total sleeping time (including naps) ？ _________

27) Do you think that cardiopulmonary diseases are associated with air pollution?

○ Yes

○ No

○ I don’t know

28) From which channel have you heard about AQI?

○ Broadcast

○ TV

○ Phone (e.g APP)

○ Computer

○ Direct measurement

○ Talking with friends

○ Own observations

○ Other ____

29) The total annual income of your family is

○ <RMB30,000

○ RMB30,000-80,000

○ RMB80,000-150,000

○ RMB150,000-800,000

○ >RMB800,000

30) Are you willing to volunteer in air pollution and human health studies?

○ Yes

○ No
