# Supplementary material for: Assessment of personal exposure to particulate air pollution: the first result of City Health Outlook (CHO) project
Source: BMC Public Health. 2019 Jun 7;19:711. doi: 10.1186/s12889-019-7022-8 (PMC6555980; doi:10.1186/s12889-019-7022-8)
Supplement: Supplementary file 4 — The physical examination procedure used in this study. (DOCX 15 kb) [file 12889_2019_7022_MOESM4_ESM.docx]

**Additional file 4.** The physical examination procedure used in this study.

Cardiorespiratory fitness is proven to be a strong indicator of cardiovascular disease and all-cause mortality (Ross et al., 2016) and is commonly diagnosed by cardiopulmonary exercise testing, which analyzes gas exchange under different physical stress levels (Reeves et al., 2018). In the test, we used MetaMax 3B CORTEX– a breath-by-breath mobile gas analyser (CORTEX Biophysik GmbH, Leipzig, Germany) to measure the full functional capacity in an objective, non-invasive way. Each subject wore a breathing mask that was connected to the measurement module of MetaMax in a chest and back carrying system. A Polar H7 Bluetooth heart rate sensor (POLAR) was tied around their check. Each session included stationary cycle-ergometer exercise (Customed bicycle ergometer ec3000, Ottobrunn, Germany) while in a zero-resistance mode for the first two minutes. The internal resistance was then adjusted to 50 watts for male and 25 watts for female and gradually increased to the limit of subject’s work intensity for 10-20 mins. All data were immediately transmitted through a Bluetooth telemetry to a PC using MetaSoft Studio software (CORTEX Biophysik GmbH, Leipzig, Germany) that supports guided intuitive workflows and standardized process for each user. The system was calibrated prior to each test. After each session, the MetaSoft Studio would perform diagnostics and identify whether the subject’s cardiopulmonary function is normal or not.

The blood pressure measurements were taken by the subjects in comfortable indoor conditions using an automatic ambulatory Omron blood pressure monitor with cuffs placed on the right upper arm. Each subject was asked to refrain from food and water and rest 15 mins in a sitting position before we took at each time point three readings with a 2-3 min pause in between using the same device. Blood pressure was determined by the mean of the three measurements.

Body mass index and other body composition parameters were measured by the research-grade Inbody 770 body composition analyzer (Inbody Co., Ltd, Republic of South Korea). The body composition describes the percentages of fat, bone, water, and muscle in human bodies and is used to determine the level of leanness and obesity. Subjects stepped on the instrument with barefoot, grabbed the handles and placed their thumbs on the oval electrodes, and kept their arms straight and away from the body for 1 min until the test was completed.

# Reference:

Reeves, T., Bates, S., Sharp, T., Richardson, K., Bali, S., Plumb, J., Anderson, H., Prentis, J., Swart, M. and Levett, D.Z.H., 2018. Cardiopulmonary exercise testing (CPET) in the United Kingdom—a national survey of the structure, conduct, interpretation and funding. Perioperative Medicine, 7(1), p.2.

Ross, R., Blair, S.N., Arena, R., Church, T.S., Després, J.P., Franklin, B.A., Haskell, W.L., Kaminsky, L.A., Levine, B.D., Lavie, C.J. and Myers, J., 2016. Importance of assessing cardiorespiratory fitness in clinical practice: a case for fitness as a clinical vital sign: a scientific statement from the American Heart Association. Circulation, pp.CIR-0000000000000461.
