# Supplementary material for: Assessment of personal exposure to particulate air pollution: the first result of City Health Outlook (CHO) project
Source: BMC Public Health. 2019 Jun 7;19:711. doi: 10.1186/s12889-019-7022-8 (PMC6555980; doi:10.1186/s12889-019-7022-8)
Supplement: Supplementary file 5 — Daily activity diary that each subject was asked to fill during the survey campaign. (DOCX 18 kb) [file 12889_2019_7022_MOESM5_ESM.docx]

**Additional file 5.** Daily activity diary that each subject was asked to fill during the survey campaign.

1) Have you been wearing the health and environmental sensors today?

○ Yes

○ No

2)

When did you go to bed last night?

When did you get up this morning?

Total sleeping time (including naps):

3) Did you wear any mask today?

○ Yes, (please specify the brand and model: _______________)

○ No

4) Which of the following transportation methods did you use to get to and from work today:

|  | Subway | Bus | Taxi or drive your own car | Electric/motorcycle | Bike | Walk |
| --- | --- | --- | --- | --- | --- | --- |
| To work | □ | □ | □ | □ | □ | □ |
| From work | □ | □ | □ | □ | □ | □ |

5) Total working hours today:

○ < 1 hour

○ 1-2 hours

○ 2-3 hours

○ 3-4 hours

○ > 4 hours

6) Your schedule of the day

| Serial Number | Starting time | Transportation/ Location | Mode of transportation/ Activity | Activity intensity |
| --- | --- | --- | --- | --- |
|  |  |  |  |  |
|  |  |  |  |  |
