# Supplementary material for: Assessment of personal exposure to particulate air pollution: the first result of City Health Outlook (CHO) project
Source: BMC Public Health. 2019 Jun 7;19:711. doi: 10.1186/s12889-019-7022-8 (PMC6555980; doi:10.1186/s12889-019-7022-8)
Supplement: Supplementary file 6 — The core modules of the CHO Platform. (DOCX 16 kb) [file 12889_2019_7022_MOESM6_ESM.docx]

**Additional file 6.** The core modules of the CHO Platform

Most parts of the recruitment process were centrally controlled by the CHO platform—an integrated management system that seamlessly combines all of the project’s systems into one complete framework, enabling different users to be assigned as different roles and functions while working as a single unit with unified objectives. The CHO platform has three core modules: the public website, the participant portal, and manager dashboard. The public website broadcasts information to the public. The participant portal allows the registration and entry of new subjects, responses to survey questions, and receiving daily reports about their exposure level and activities. It adopts the responsive web design that the subjects’ user interface can render well on a variety of devices. Finally, the manager dashboard functions as the control panel that supports real-time monitoring of device activity and pushes automatic notifications to offline, abnormal, or low-battery devices. It manages all environmental and GPS data that are wirelessly transferred to the backend server. In addition, it contains a survey administration interface for survey creation, data collection, and visualization of responded questionnaire data in statistical figures and maps.
